# Profiling *β* Thalassemia Mutations in Consanguinity and Nonconsanguinity for Prenatal Screening and Awareness Programme

**DOI:** 10.1155/2015/625721

**Published:** 2015-10-21

**Authors:** Ravindra Kumar, Vandana Arya, Sarita Agarwal

**Affiliations:** ^1^Central Research Laboratory, Sri Aurobindo Medical College and PG Institute, Indore, Madhya Pradesh 453111, India; ^2^Department of Molecular Hematology, Sir Ganga Ram Hospital, New Delhi 110060, India; ^3^Department of Genetics, Sanjay Gandhi Post Graduate Institute of Medical Sciences, Lucknow, Uttar Pradesh 226014, India

## Abstract

Mutation spectrum varies significantly in different parts and different ethnic groups of India. Social factors such as preference to marry within the community and among 1st degree relatives (consanguinity) play an important role in impeding the gene pool of the disease within the community and so in society by and large. The present paper discusses the role of consanguinity in profiling of beta thalassemia mutation, and thus the approach for prenatal screening and prevention based awareness programme. Clinically diagnosed 516 cases of beta thalassemia were screened at molecular level. A detailed clinical Proforma was recorded with the information of origin of the family, ethnicity, and consanguinity. The present study reports that subjects originating from Uttar Pradesh, Uttarakhand, Bihar, and Jharkhand have c.92+5G>C and c.124_127delTTCT mutation as the commonest mutation compared to the subjects hailing from Madhya Pradesh and Chhattisgarh and Nepal where sickle mutation was found more common. In 40 consanguineous unions more common and specific beta mutations with higher rate of homozygosity have been reported. This consanguinity-based data helps not only in deciding target oriented prenatal diagnostic strategies but also in objective based awareness programmes in prevention of thalassemia major birth.

## 1. Introduction

Thalassemia is a heterogeneous group of hemoglobin disorders in which the production of normal hemoglobin is partly or completely suppressed as a result of the defective synthesis of one or more globin chains [1]. Thalassemia presents a significant health problem in 71% of 229 countries, and these 71% of countries include 89% of all births worldwide. It has been estimated that approximately 7% of the world population are carriers of thalassemia and about 56,000 have a major thalassemia, including at least 30,000 who need regular transfusions to survive and 5,500 who die perinatally due to *α* thalassemia major [2].

This increase of thalassemia birth might be due to lack of prevention and awareness programs running in India. Awareness strategies need intervention at two levels, that is, among medical practitioners and population in mass.

Awareness programs are usually less popular in mass as there exist variable ethnic groups and various customs pertaining to their marriage pattern within the community itself prevent their exposure to wide society and prevent them from coming out of the traditions existing within the community. This by and large affects their decision in making mating pattern. The marriage within the community or relatives is relatively more or less dictated by their religious gurus and then it becomes very hard to overcome rituals, as education also becomes a limiting factor to understand the facts of life with quality. As a result, the country gets overburdened with the genetic disorders and family faces the social, mental, and economical trauma due to limited medical facilities available in the state.

Prevention of the disease by genetic counseling and prenatal diagnosis has an ultimate option in those parts of the world where limited resources for the medical care persist.

Pattern of mutations varies significantly between different geographical regions and community/ethnic groups; however each population and ethnic group has its own sets of common mutations [3–6]. This knowledge of spectrum of mutations enables medical fratinity and nongovernment organizations (NGOS) to create awareness programs, genetic counseling, and target oriented prenatal diagnosis to avoid long awaited trauma to the high risk couples [7].

In India the social factors such as preference to marry within the community and among 1st degree relatives (consanguinity) is quite prevailing and thus plays an important role in increasing the gene pool of the mutation responsible for disease within the community [5, 8, 9]. In that scenario target oriented prenatal diagnosis helps early detection of the fetus status.

Therefore, our aim of study is to first dissect the regional and ethnic profiling of beta thalassemia mutation and further look into the angle of the implication of consanguinity on spectrum of mutation in the state of Uttar Pradesh which is the second largest state of the country and where social and casts based marriages are very common.

## 2. Material and Methods

Total 516 clinically and molecularly diagnosed cases of beta thalassemia were included in the present study. The data pertaining to origin of the family, ethnicity, and consanguinity were evaluated for making reasonable and sizable group for including in the study. All the patients were grouped according to their origin and community. For beta thalassemia mutation detection analysis, 2 mL of blood was drawn and collected in EDTA coated vials from each individual. DNA was extracted from peripheral blood leucocytes by commercial available DNA extraction kit (Qiagen). Mutation detection was done using ARMS-PCR as described previously by Agarwal et al. 2000 [3]. Data was entered in Microsoft Excel 2007 and spectrum of mutation was identified in different groups.

## 3. Results

**Figure 1:**
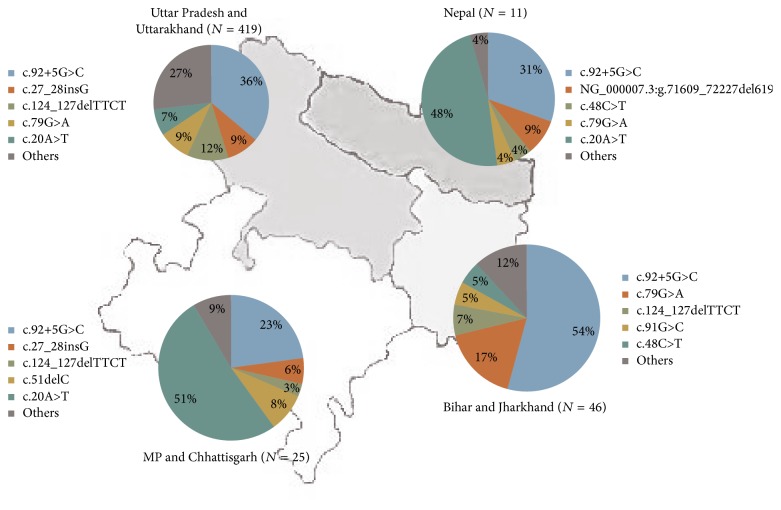
Spectrum of beta mutations.

These 516 cases were grouped according to their native place. The 419 subjects were from Uttar Pradesh (UP) and Uttarakhand, 46 were from Bihar and Jharkhand, 25 were from Madhya Pradesh (MP) and Chhattisgarh, and 15 were the natives of Nepal. The remaining 11 cases were from other states. Spectrum analysis in different neighboring states or country shows quite different patterns from each other. Interestingly, the high frequency of sickle mutation (c.20A>T) was found in the subjects who had family origin from MP and Chhattisgarh and Nepal (Figure 1).

Total 674 chromosomes from 516 individuals were analyzed to find out molecular lesions of beta globin gene. To assess the mutation pattern among Hindus and Non-Hindus communities as their marriage rituals differ from each other, we have divided subjects into two groups (Table 1). A quite variable pattern was observed in both the groups. c.48C>T mutation was found with high frequency of 8.8% in Non-Hindus compared to 1.9% in Hindus.

Since the marriage pattern and the customs prevailing within the community are different and so is the pattern of mutation, we have divided the group into two, depending upon the consanguinity and nonconsanguinity in the family.  Table 2 summarizes the pattern of beta mutations in consanguineous versus nonconsanguineous group. Since we have not observed any consanguinity in sickle cell (c.20A>T) and HbE (c.79G>A) group, we have excluded those 126 variant cases from Table 2.

This present compiled data showed 40 consanguineous families. Interestingly only 7 beta mutations covered the whole consanguineous group; however, 11 beta mutations are attributed to the nonconsanguineous group (Table 2).

c.92+5G>C was the most common mutation in both the groups. In consanguineous group c.27_28insG, c.48C>T, and c.124_127delTTCT followed it whereas in nonconsanguineous group c.124_127delTTCT, c.51delC, 619 bp, and c.27_28insG mutations were common after c.92+5G>C. Approximately 80% of the chromosomes from consanguineous category were covered by only 3 mutations whereas 8 mutations are needed to screen the nonconsanguineous group. A more heterogeneity in the nonconsanguineous group was found as compared to consanguineous group (Table 2).

In order to find out the homozygosity pattern for these mutations, we observed an increased rate of homozygosity (15%) in consanguineous group as compared to nonconsanguineous group (6.3%) (Table 3).

## 4. Discussion

Prevention of *β* thalassemia by genetic counseling and prenatal diagnosis is an important health issue in India. One of the approaches for community control of *β* thalassemia, outlined by the World Health Organization (WHO), is documentation of molecular heterogeneity of the disease. The knowledge of the geographic profiling of mutation in the population has proved to be the best strategy in diagnosis of the disease at prenatal level.

Several previous reports from North India have emphasized the need for regular evaluation of the mutation, as the shift in the frequencies of the mutation is very high [5, 6, 8, 9]. Thereby we have analyzed the spectrum of mutation in different neighboring states or country. This data indicates that few mutations are common in different parts of the country, though the frequency differs (Figure 1). In Uttar Pradesh and Uttarakhand, Bihar and Jharkhand the c.92+5G>C mutation was found in 36% and 54%, respectively. The HbS, a common Hb variant, was found more frequently in Madhya Pradesh (51%) and Nepal (48%). Surprisingly c.92+1G>T, one of the common mutations reported in Punjabis by Garewal and Das in 2003 [10], was found to be absent in Madhya Pradesh, Bihar and Jharkhand, and Nepal.

This study clearly indicates that consanguineous marriages have a higher risk of producing affected offspring than general populations due to high gene pool. The consanguineous marriages are frequently present in South India as compared to North India [11, 12]. However no specific report related to the overall prevalence of consanguinity in India has been reported so far; Bittles in 2002 has reported the prevalence of consanguineous marriages in different parts of India which was basically compiled by National Family and Health Survey (IIPS 1995) in 1992-93 [13].

As our centre is the referral centre for genetic disorders, the majority of the cases are from Uttar Pradesh, Uttarakhand, Bihar and Jharkhand, Madhya Pradesh and Chhattisgarh, and neighboring country Nepal. In our hospital based study we have found that consanguinity is 7.9% in Uttar Pradesh and Uttarakhand, while in Madhya Pradesh and Chhattisgarh it is 1.6% and 4.3% in Bihar and Jharkhand. The present data supports the findings of Bittles as they have reported the rate of consanguineous marriages to be 7.5, 4.1, and 5% in Uttar Pradesh and Uttarakhand, Madhya Pradesh and Chhattisgarh, and Bihar and Jharkhand, respectively [13].

When focusing on consanguinity rate by religion, Bittles has reported a remarkably higher frequency in Muslims (59.6%) than in Hindus (1.7%) and other religious groups [13, 14]. In our data we have not observed the consanguinity in other religions like Christians, Buddhists, and nonbelievers.

The highest rates of consanguineous marriage in North Central part of India are usually reported from rural areas and among the economically weak and least educated groups. Considerable attention is paid to the role of consanguineous marriages as a causative factor in the high prevalence of genetic disorders [15–17]. At the same time the potential influence of this community endogamy on disease type, its severity, and the approach towards management has not been noticed properly and has been underestimated.

The probability of homozygosity rate in consanguineous marriages is higher than that of unrelated parents; this may be due to the limited gene pool and thus more expression of recessive alleles. The statement is supported by the present study as homozygosity was found significantly higher (15%) in consanguineous group as compared to nonconsanguineous group (6.3%).

Thus consanguinity causes clustering of mutations within the community, which increases the risk of a thalassemic child to be born.

This study shows that in a consanguineous group the specific set of mutations accounts for nearly 80% of subjects. This prompted the molecular geneticist to extend the approach for the early and rapid prenatal diagnosis and development of diagnostic kit with few selected mutations for the consanguineous unions on the one hand and strategy of awareness programmes for NGOs on the other hand.

## Figures and Tables

**Table 1:** Spectrum of mutation in Hindu and Non-Hindu groups.

Mutations	Chromosomes	
	Non-Hindus [% prevalence]	Hindus [% prevalence]
c.92+5G>C	36 [31.8]	216 [38.5]
c.124_127delTTCT	12 [10.6]	57 [10.1]
c.27_28insG	13 [11.5]	40 [7.1]
c.92+1G>T	1 [0.9]	22 [3.9]
NG_000007.3:g.71609_72227del619	1 [0.9]	35 [6.2]
c.51delC	5 [4.4]	31 [5.5]
c.91G>C	5 [4.4]	21 [3.7]
c.-50A>T	2 [1.7]	15 [2.6]
c.48C>T	10 [8.8]	11 [1.9]
c.92+1G>A	1 [0.9]	2 [0.3]
c.-138C>T	—	4 [0.7]
c.79G>A	15 [13.2]	46 [8.1]
c.20A>T	11 [9.7]	64 [11.4]

	113	561

**Table 2:** Status of the *β* mutation in consanguineous and nonconsanguineous group.

Mutations	Consanguineous [ch = 52] [%]	Nonconsanguineous [ch = 388] [%]
c.92+5G>C	19 [36.5]	168 [43.3]
c.124_127delTTCT	5 [9.6]	49 [12.6]
c.27_28insG	15 [28.8]	29 [7.5]
c.48C>T	7 [13.4]	12 [3.1]
c.51delC	—	44 [11.3]
c.92+1G>T	1 [1.9]	16 [4.1]
NG_000007.3:g.71609_72227del619	3 [5.7]	30 [7.7]
c.91G>C	—	23 [5.9]
c.-50A>T	2 [3.8]	10 [2.6]
c.92+1G>A	—	3 [0.7]
c.-138C>T	—	4 [1.0]

ch = chromosomes.

**Table 3:** Homozygosity versus compound heterozygosity in two groups.

	Consanguineous [9]	Nonconsanguineous [85]
Homozygous	6 (15%)	23 (6.3%)
Compound heterozygous	3 (7.5%)	62 (17.1%)
